# Rapid and Sensitive One-Tube Detection of Getah Virus Using RT-LAMP Combined with *Pyrococcus furiosus* Argonaute

**DOI:** 10.3390/vetsci12020093

**Published:** 2025-01-24

**Authors:** Zhong Liu, Fosheng Yang, Mengtao Fang, Qi Wu, Ke Fan, Dongyan Huang, Yu Ye, Gen Wan, Deping Song

**Affiliations:** 1 Department of Preventive Veterinary Medicine, College of Animal Science and Technology, Jiangxi Agricultural University, Nanchang 330045, China; 19970042875@163.com (Z.L.); 15989992759@163.com (F.Y.); fmt15797711231@126.com (M.F.); wuqi3950@163.com (Q.W.); fanke1112@163.com (K.F.); huangdongyan@jxau.edu.cn (D.H.); yeyu@jxau.edu.cn (Y.Y.); 0000005341@jxau.edu.cn (G.W.); 2 Jiangxi Engineering Research Center for Animal Health Products, Jiangxi Agricultural University, Nanchang 330045, China

**Keywords:** Getah virus, RT-LAMP, *Pyrococcus furiosus* Argonaute, virus detection

## Abstract

Getah virus (GETV) is a mosquito-borne virus that poses a significant threat to both animal and public health. Traditional diagnostic methods for GETV, such as RT-PCR and RT-qPCR, require expensive equipment and complex procedures, making them unsuitable for rapid, on-site detection. The combination of RT-LAMP and P*f*Ago offers a novel approach for nucleic acid detection, providing high specificity and effective without the need for sophisticated instruments. Herein, we developed a RT-LAMP combined with P*f*Ago assay for GETV detection. The RT-LAMP assay was conducted at 60~65 °C, and then the RT-LAMP product was cleaved, together with a fluorescent probe, mediated by P*f*Ago at 95 °C. After optimizing the primary reaction conditions, the detection limit of the RT-LAMP-P*f*Ago assay was 100 copies/µL. Importantly, there was no cross-reactivity with other viruses, including PEDV, PDCoV, PoRV, PRRSV, and CSFV. Compared to qPCR, analysis of 86 clinical samples showed that LAMP-P*f*Ago had a consistent positive rate with the qPCR method. In conclusion, we developed a valuable diagnostic tool for the rapid detection of GETV, enabling timely surveillance and control measures to mitigate the impact of GETV outbreaks.

## Simple Summary

Getah virus (GETV) is a zoonotic virus transmitted by mosquitoes. Various mammals are naturally infected with GETV, including foxes, pigs, horses, cattle, and sheep. To develop and validate a rapid, sensitive, and portable one-tube assay for the detection of GETV, we employed reverse transcription loop-mediated isothermal amplification (RT-LAMP) in conjunction with *Pyrococcus furiosus* Argonaute (P*f*Ago) to provide a straightforward and precise method for detecting porcine GETV in this study. LAMP is used to specifically amplify the target gene of GETV, after which P*f*Ago is directed to cleave the target gene and the probe, releasing a fluorescent signal. Additionally, the assay results can be observed under UV light or quantified using a fluorescence quantitative instrument. Our experiments demonstrate that this assay is highly specific, capable of detecting extremely low levels of the virus without cross-reacting with other viruses. When tested on real clinical samples, our novel method performed comparably to the gold standard quantitative real-time PCR test. This study provides a rapid, accurate, efficient, portable, and low-cost nucleic acid diagnostic method for the detection of GETV, which could serve as a powerful tool for the prevention and control of GETV.

## 1. Introduction

Getah virus (GETV) is a neglected mosquito-borne virus belonging to the *Alphavirus* genus and *Togaviridae* family. Based on viral antigens, GETV is classified under the Semliki Forest virus complex [1]. GETV is an enveloped, single-stranded, positive-sense RNA virus with a genome of approximately 11.2 kb [2]. The genomic RNA of GETV contains two large open reading frames (ORFs) that encode the four nonstructural proteins (nsP1, nsP2, nsP3, and nsP4) and five structural proteins (capsid [C], E3, E2, 6K, and E1) [3]. The prototype strain MM2021 was the first GETV strain isolated from *Culex gelidus* collected in Malaysia in 1955. Currently, GETV is widespread in 13 Asian and pan-Pacific countries, including China, South Korea, Japan, Russia, the Philippines, Malaysia, and Australia [4]. Sero-epizootiological studies have demonstrated that GETV has a broad host spectrum, with natural infections in mosquitoes and animals including pigs, horses, goats, cattle, boars, blue foxes, and humans, suggesting a significant expansion in the virus’s host range [5,6,7,8,9,10]. As a multi-host pathogen, GETV is becoming increasingly serious and poses a substantial threat to animal safety and public health. To date, GETV has been found to be pathogenic to horses, pigs, and cattle, causing reproductive disorders, fever, neurological symptoms, diarrhea, and death in mammals, resulting in significant economic losses in the livestock industry [10,11]. Horses infected with GETV often exhibit clinical signs such as fever, rash, and leg edema [12]. Infected piglets display symptoms including arthritis, fever, anorexia, diarrhea, tremors, hind limb paralysis, ataxia, and high mortality. Pregnant sows infected with GETV may experience reproductive disorders such as spontaneous abortion and stillbirth [13]. Therefore, rapid diagnosis is crucial for early surveillance and control to mitigate the risk of GETV outbreaks.

The current major methods of GETV detection include ‘golden standard’ methods such as virus isolation, virus neutralization tests, and indirect fluorescent antibody assay, as well as molecular technologies like RT-PCR, RT-qPCR, loop-mediated isothermal nucleic acid amplification (LAMP), and RT-recombinase aided amplification (RAA). Serological assays like the enzyme-linked immunosorbent assay (ELISA) are also commonly used, along with novel methods such as CRISPR-based technologies [14,15,16,17,18]. However, these processes are not only time-consuming but also demanding on both laboratories and laboratorians. Serological diagnostics and ELISA face similar challenges, including complexity and lack of timeliness [19,20]. With advancements in molecular diagnostic techniques, RT-PCR [21] and RT-qPCR [22] are beginning to be applied to the diagnosis of GETV. Nevertheless, these methods require sophisticated equipment, making them difficult to implement in resource-limited areas. While emerging isothermal amplification techniques like RAA [16] and LAMP [17] show promise in addressing this issue, they still struggle with non-specific amplification. As a result, many studies are turning to CRISPR technology with isothermal amplification to mitigate the false positive results of amplification [23]. However, challenges arise due to constraints in target PAM sequences.

Argonaute (Ago) proteins, widely distributed across many biological systems, have been reported to possess potential in nucleic acid detection [24]. P*f*Ago, an Argonaute protein derived from *Pyrococcus furiosus*, can utilize 5′ phosphorylated short ssDNA to specifically cleave both DNA and RNA targets [25]. Unlike CRISPR, P*f*Ago does not depend on PAM sequences for target recognition. In contrast to CRISPR-associated (Cas) nucleases, which require long RNA guides, P*f*Ago, as well as most prokaryotic Ago proteins, employs short DNA molecules as a guide DNA (gDNA). Furthermore, short gDNA is more cost-effective to synthesize and easier to store, which promotes the development of Ago-based nucleic acid detection [26]. P*f*Ago’s systematic cleavage has been exploited for specific detection of various viruses, including SARS-CoV-2, influenza viruses, PEDV, and PDCoV [27,28,29,30].

Previous reports have demonstrated P*f*Ago’s potential for rapid and on-site detection of viruses in conjunction with RTX-PCR, RAA, and RPA [30,31,32]. In this study, we developed a one-tube method for detecting GETV that combines LAMP with the P*f*Ago system. This method can accurately detect GETV within 60 min and demonstrates ultra-high sensitivity and strong specificity. It offers a rapid, reliable, convenient, and cost-effective solution for detecting GETV without expensive equipment.

## 2. Materials and Methods

### 2.1. Sample Collection and Viruses

A total of 86 clinical samples, including intestinal, liver, and lung tissues, were collected from pig farms in Jiangxi Province, China. The isolated viruses, including GETV, porcine epidemic diarrhea virus (PEDV), porcine deltacoronavirus (PDCoV), porcine rotavirus (PoRV), porcine reproductive and respiratory syndrome virus (PRRSV), and classical swine fever virus (CSFV), were used to establish the LAMP-P*f*Ago method. The genomic RNAs of these viruses were extracted using RNAiso Plus (Takara, Beijing, China) following the manufacturer’s instructions, and cDNAs were synthesized using the PrimeScript RT Master Mix (Takara, Beijing, China).

### 2.2. RT-LAMP Assay

To identify the conserved regions, the complete genome sequences of GETV currently available in GenBank were analyzed by MEGA11 (https://www.megasoftware.net/, accessed on 10 March 2024). The conserved sequence fragment of the NSP1 gene of GETV was selected as the targeted region, and used to design the GETV LAMP primers with PrimerExplore (http://primerexplorer.jp/elamp4.0.0/index.html, accessed on March 12 March 2024). GETV-F3/B3 were the forward outer/reverse outer primers, GETV-FIP/BIP were the forward inner/reverse inner primers, and GETV-LB was the loop primer used for LAMP amplification. The gDNA design was based on P*f*Ago’s ability to cleave DNA complementary to its bases, mediated by ssDNA with a phosphate group at the 5′*-*end. Moreover, the probe was designed as an ssDNA sequence complementary to the newly generated gDNA, flanked by a carboxyfluorescein (FAM) at the 5′-end and a quencher BHQ1 at the 3′-end termini. The qPCR-F/R was utilized for quantitative real-time PCR target GETV (Table 1).

In order to construct the standards of GETV NSP1 gene, the targeted cDNA of GETV underwent PCR-based amplification with the primer GETV-NSP1-F/R. The PCR parameters were as follows: 98 °C for 5 min, 35 cycles of 98 °C for 10 s, 58 °C for 20 s, 72 °C for 30 s, and a final extension at 72 °C for 5 min. The resulting product was purified with the E.Z.N.A™ Gel Extraction Kit (Omega, Suzhou, China), ligated into pMD™ 19-T (Takara, Beijing, China) to form a standard plasmid, and then cloned into *E. coli*. The recombinant plasmid was extracted using the TIANprep Mini Plasmid Kit (TIANGEN, Beijing, China) following the protocol of the manufacturer. The concentration of the plasmid DNA was determined by the NanoDrop 2000 spectrophotometer (Thermo Fisher Scientific, Waltham, MA, USA), and the plasmid copy number was calculated based on the previous method [33].

The LAMP reaction was carried out in a final volume of 25 μL. To optimize the reaction conditions, the reactions contained different concentrations of MgSO_4_ (at 2, 4, 6, 8, and 10 mM, Sigma-Aldrich, St. Louis, MO, USA), dNTPs mix (at 0.2, 0.4, 0.6, 1.0, and 1.4 mM, Sangon, Shanghai, China), each of inner primer GETV-FIP and GETV-BIP (at 0.2, 0.4, 0.8, 1.2, 1.6, 2.0 μM), each of outer primer GETV-F3 and B3 (0.2 μM), loop primer GETV-LB 0.4 μM, 2.5 μL 10× Isothermal Amplification Buffer, *Bst* 2.0 DNA Polymerase (8U, New England Biolabs, Ipswich, MA, USA), and 1 μL template DNA from standard plasmid or cDNA from clinical samples. Furthermore, the temperature of the LAMP reaction was determined by incubating the reaction systems at 53, 57, 61, 65, and 70 °C for 50 min in a water bath, respectively. The reaction time was then determined at 10, 20, 30, 40, and 50 min, respectively. The LAMP products were electrophoresed on 1.5% agarose gel in 1× TAE buffer, or directly visualized by adding 1× SYBR Green I in the reaction tube for diction by a color change.

### 2.3. PfAgo Cleavage

The P*f*Ago endonuclease was purchased from Jiaohong Biotech (Shanghai, China). The LAMP primers, gDNAs, and probes were synthesized by Sangon Biotech (Shanghai, China) and are listed in Table 1. To integrate the P*f*Ago system into the RT-LAMP assay, 3 μL of the abovementioned LAMP reaction product was transferred to P*f*Ago reaction with 20 μL volume containing 2 μL of 10× reaction buffer, 1.5 mM Mn^2+^, 0.75 μM of each gDNA (total three gDNAs), 4 μL P*f*Ago (200 U/μL), and 0.25 μM probe with FAM at the 5′ end and BHQ1 at the 3′ end on the top. The reaction was carried out in a real-time fluorescence quantitative PCR instrument (CFX96TM, BioRad, Hercules, CA, USA) at 95 °C for 30 min for the cleavage and the fluorescence signal following the P*f*Ago-mediated cleavage reaction was observed under UV light.

### 2.4. One-Tube RT-LAMP-PfAgo Assay Development

To combine the RT-LAMP and P*f*Ago reactions into a single tube, the LAMP reaction was conducted as follows: 2.5 μL 10× Isothermal Amplification Buffer, 1.0 mM dNTP mix, 6 mM MgSO_4_, 1 μL Bst 2.0 DNA Polymerase, a primer mixture (1.6 μM FIP, 1.6 μM BIP, 0.2 μM F3, 0.2 μM B3, and 0.4 μM LB), and 2 μL sample cDNA. The LAMP reaction mixture was introduced to the bottom of the tube, followed by 10 μL of the P*f*Ago system consisting of 2 μL of 10× reaction buffer, 1.0 mM Mn^2+^, 2.0 μM of each gDNA, 3 μL P*f*Ago (200 U/μL), and then 0.25 μM probe wasplaced in a filter paper disc attached to the tube cap. After incubating for 30 min at 65 °C, the mixture was centrifuged at 5000× *g* for 1 min, allowing the filter paper disc to fall into the bottom of the tube, where it was thoroughly mixed and incubated for an additional 30 min at 95 °C. The reaction results were observed in a fluorescence detector or under UV light.

To test the sensitivity, specificity, and detection limit of the established assay, the recombinant standard plasmid of GETV NSP1 gene was serially diluted from 1 × 10^6^ copies/μL to 1 × 10^0^ copies/μL and used as templates to assess the sensitivity of the LAMP-P*f*Ago assay, and the genomic cDNA of PEDV, PDCoV, PoRV, PRRSV, and CSFV was utilized to evaluate the specificity of the LAMP-P*f*Ago system. The results were observed under UV light after 30 min of the LAMP-P*f*Ago reaction at 95 °C.

### 2.5. Validation and Field Testing

Total RNA from clinical samples was extracted using RNAiso Plus (Takara, Beijing, China) and cDNAs were synthesized using the Reverse Transcriptase M-MLV (Takara, Beijing, China). The cDNAs from these samples were then used for GETV screening both using RT-LAMP-P*f*Ago and RT-qPCR [10]. The clinical application was evaluated by comparing the detection results of RT-LAMP-P*f*Ago with those of RT-qPCR.

### 2.6. Statistical Analysis

Each of the experiments was carried out in triplicate and GraphPad Prism was used to statistically evaluate the data. A students’ *t* test was employed to analyze the data, with *p*-values of less than 0.05 considered statistically significant.

## 3. Results

### 3.1. Principle of the LAMP-PfAgo Method

**Figure 1:**
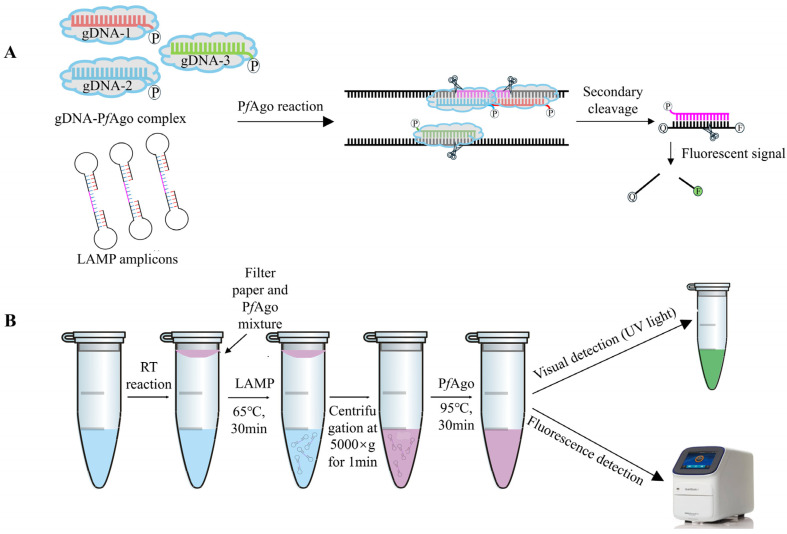
RT-LAMP-P*f*Ago-based GETV Detection Methods. (**A**) Schematic diagram of the LAMP-P*f*Ago assay designed to produce sequence-specific fluorescent signals. (**B**) Schematic workflow of the one-tube LAMP-P*f*Ago process for detecting GETV.

The mechanism underlying the LAMP-P*f*Ago assay for GETV is illustrated in Figure 1. Briefly, LAMP-amplified fragments are specifically identified by the P*f*Ago protein guided by 5′-phosporylated gDNA. P*f*Ago then cleaves the phosphodiester bonds between the 10th and 11th bases of the target DNA from the 5′ end upon base pairing between gDNA and one strand of the LAMP amplicons, resulting in a new 5′-phosporylated ssDNA. This newly formatted ssDNA can act as new gDNA, specifically identifying the synthetically designed single-stranded DNA probe labeled with a fluorophore (FAM) and a quencher (BHQ1) at its termini. P*f*Ago then cleaves the DNA probe to release fluorescent signals (Figure 1A). For one-tube LAMP-P*f*Ago detection, viral RNA is extracted and cDNA is synthesized, serving as the template for the LAMP reaction. The P*f*Ago mixture is placed in a filter paper disc attached to the inside of the tube cap. The LAMP reaction is carried out at 65 °C for 30 min. Subsequently, the reaction tube is centrifuged at 5000× *g* for 1 min, allowing the filter paper disc to fall to the bottom of the tube. The P*f*Ago reaction mixture is then thoroughly mixed with the LAMP products and incubated at 95 °C for 30 min. Fluorescent signals can be detected using a fluorescence detector or visualized with the naked eye under UV light (Figure 1B).

### 3.2. Optimization of LAMP Assay for Detection of GETV

**Figure 2:**
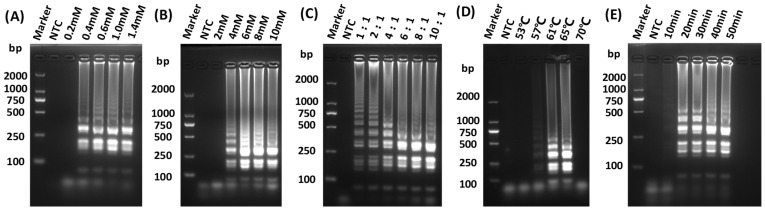
Optimization of LAMP reaction conditions. (**A**) Screening for optimal concentration of dNTPs for LAMP; (**B**) Screening for optimal concentration of MgSO_4_ for LAMP; (**C**) Screening for optimal reaction internal and external primer concentration ratio for LAMP; (**D**) Screening for optimal reaction temperature for LAMP; (**E**) Screening for optimal reaction times for LAMP.

The LAMP assay for detecting GETV was successfully developed. To determine the optimal amplification efficiency for isothermal amplification, reactions were prepared with varying concentrations of dNTPs mix, MgSO_4_, and each of inner primers FIP and BIP. Furthermore, the temperature of the LAMP reaction was determined by incubating the reaction systems at 53, 57, 61, 65, and 70 °C for 10, 20, 30, 40, and 50 min in a water bath, respectively. The optimized parameters of the reaction system were as follows: 1 mM dNTPs, 6 mM MgSO_4_, and an internal to external primer ratio of 8:1 (Figure 2A–C), with an incubation condition at 65 °C for 30 min in a water bath (Figure 2D,E).

### 3.3. Optimization of LAMP-PfAgo Reaction

**Figure 3:**
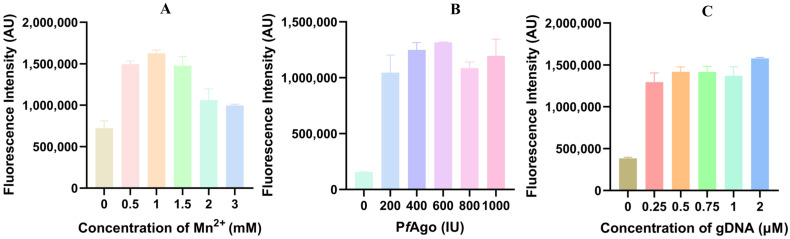
Optimization of primary reaction components for LAMP-P*f*Ago. (**A**) Fluorescence intensity measured by LAMP-P*f*Ago reaction at different Mn^2+^ concentrations; (**B**) Fluorescence intensity measured by LAMP-P*f*Ago reaction at different P*f*Ago concentrations; (**C**) Fluorescence intensity measured by LAMP-P*f*Ago reaction at different gDNA concentrations.

To improve the efficiency of the LAMP-P*f*Ago reaction, the concentrations of Mn^2+^ (at 0, 0.5, 1.0, 1.5, 2.0, 3.0 mM), P*f*Ago (200 U/μL, at 0, 1.0, 2.0, 3.0, 4.0, 5.0 μL), and gDNA (at 0.25, 0.5, 0.75, 1.0, 2.0 μM), were optimized. The results indicated that the highest fluorescence value was achieved with 1.0 mM Mn^2+^, 600 U P*f*Ago, and 2 μM gDNA, respectively (Figure 3A–C).

### 3.4. Sensitivity and Specificity of the LAMP-PfAgo

**Figure 4:**
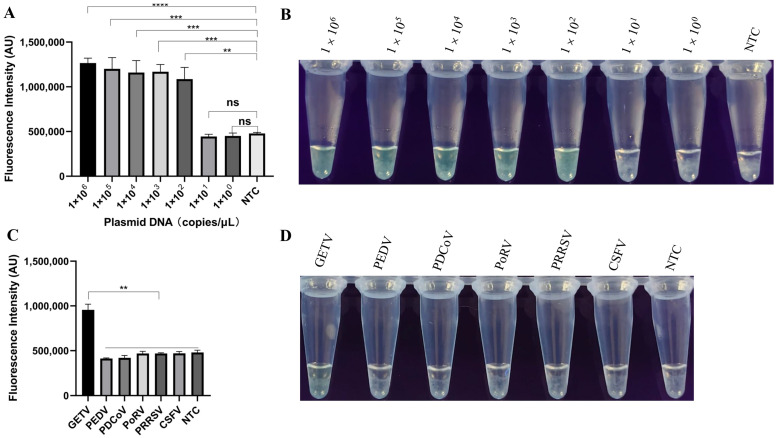
Evaluation of the sensitivity and specificity of LAMP-P*f*Ago for GETV detection. (**A**) End-point fluorescence intensity of LAMP-P*f*Ago sensitivity; (**B**) Visualization of LAMP-P*f*Ago sensitivity under UV light; (**C**) Fluorescence intensity during the specificity test for detecting GETV using LAMP-P*f*Ago; (**D**) Visualization results of LAMP-P*f*Ago specificity under UV light. Each experiment was performed in triplicate and statistical analysis was conducted using a student’s *t*-test in GraphPad Prism 8. NTC indicates negative control; ** indicates *p* < 0.01; *** indicates *p* < 0.001; **** indicates *p* < 0.0001; ns indicates no significance.

The recombinant plasmid was diluted tenfold (from 1 × 10^6^ to 1 × 10^2^ copies) and used to determine the limit of detection of the LAMP-P*f*Ago. As shown in Figure 4A, the lowest detection limit of LAMP-P*f*Ago was 1 × 10^2^ copies/μL and the results were validated under UV light (Figure 4B). Using the LAMP-P*f*Ago method constructed in this study, we detected several associated swine pathogens, including PEDV, PDCoV, PoRV, PRRSV, and CSFV. The results indicated that only GETV showed a significant increase in fluorescence during amplification (Figure 4C). The same results were visually confirmed under UV light (Figure 4D).

### 3.5. Validation and Clinical Sample Detection

**Figure 5:**
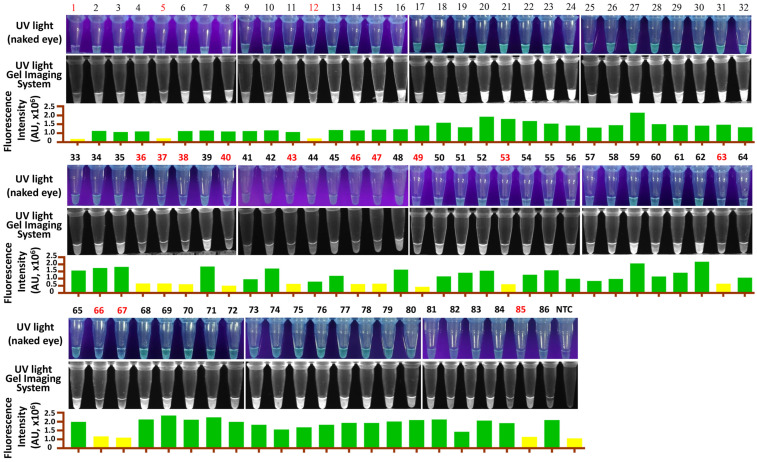
Detection results of clinical samples for GETV using the RT-LAMP-P*f*Ago assay. Samples in red indicate a negative result. NTC, negative control.

RT-LAMP-P*f*Ago and RT-qPCR were used to analyze 86 samples from pigs suspected of GETV infection. The results showed that RT-LAMP-P*f*Ago detected 70 positive samples out of 86 (Figure 5) with a 100% consistency with RT-qPCR results (Table 2). Our findings indicate that the RT-LAMP-P*f*Ago method developed in this study is suitable for clinical diagnosis.

## 4. Discussion

Since its discovery in Hainan province, China, in 1964, GETV has spread to 17 provinces [34]. As an arbovirus, GETV can infect various mammals, including blue foxes, pigs, horses, cows, and sheep [16]. Notably, antibodies against GETV have also been found in the blood serum of febrile patients [35]. Research has shown that GETV can infect human and primate cells as well [10,36]. These findings suggest that GETV poses a potential threat to public health. In pigs, GETV has been found to infect pigs in different stages. Piglets infected with GETV display symptoms such as arthritis, fever, anorexia, diarrhea, tremors, hind limb paralysis, ataxia, and high mortality. Pregnant sows infected with GETV can experience reproductive disorders, including spontaneous abortion and stillbirth [13]. Since 2015, GETV outbreaks have occurred continuously in various provinces of China, mainly causing death and/or reproductive disorders [10,11]. Sero-epidemiology investigations indicated frequent infections of GETV in swine in China [18]. However, the lack of effective vaccines or treatments results in significant economic losses to the pig industry caused by GETV infections. Enhancing early detection and surveillance of the pathogen and improving the sensitivity of detection methods are essential for the diagnosis, prevention, and control of the disease.

To date, several tools have been developed for the detection of GETV, such as RT-PCR, RT-qPCR, LAMP, RAA, and ELISA [14,15,16,17,18]. RT-qPCR is regarded as the gold standard for viral diagnosis. Although RT-PCR and RT-qPCR assays are widely used in GETV diagnosis, they are limited by requiring expensive instruments and complex reaction processes, and are thus not suitable for rapid portable clinical detection. Therefore, there is an urgent need to develop a rapid, accurate, sensitive, portable, and low-cost GETV nucleic acid diagnostic tool to meet the current demand for on-site screening and clinical diagnosis.

The P*f*Ago system offers a novel perspective for the development of nucleic acid molecular diagnostics due to its exceptional ability to recognize and cleave target DNA. Similar to the CRISPR system, P*f*Ago requires guidance from gDNA (phosphorylated short single-stranded DNA) to effectively cleave target DNA sequences [25]. Previous studies have developed a series of diagnostic methods in combination with isothermal amplification techniques, such as RAA-P*f*Ago [29], RPA-P*f*Ago [37], and LAMP-P*f*Ago [38]. The integration of the P*f*Ago system into the RT-LAMP assay can effectively prevent false positive results caused by the amplification process, thereby ensuring the accuracy of the diagnostic outcomes due to its step-by-step cleavage specificity [39,40,41]. Unlike RT-PCR and RT-qPCR, the entire reaction of RT-LAMP-P*f*Ago is maintained at two consecutive temperatures, significantly reducing the reliance on costly instruments.

In the LAMP-P*f*Ago assay developed in this study, the gDNA and probe sequences were specifically designed between FIP and BIP of the LAMP primer set. This design ensures that all the sequences recognized by P*f*Ago are derived from the amplification of target sequences, rather than from non-specific amplification in the LAMP reaction. The entire reaction is maintained at two consecutive temperatures and the results can be visualized under UV light, with a minimum detection of 10^2^ copies/µL. There was no cross-reactivity with other pig-related pathogens, demonstrating the potential of this method for detecting GETV in the field. Furthermore, the design of the single-tube operating method not only minimizes the risk of open-cap contamination but also enhances the portability and ease of use of the one-tube assay, making it suitable for deployment in resource-limited settings.

Overall, this study provides a valuable diagnostic tool for the rapid and sensitive detection of GETV, enabling timely surveillance and control measures to mitigate the impact of GETV outbreaks. The one-tube assay’s portability and ease of use make it suitable for deployment in resource-limited settings, contributing to improved public and animal health.

## 5. Conclusions

Our study underscores the significant threat posed by GETV to both animals and public health. Since its first discovery in Hainan province, China, in 1964, GETV has spread to 17 provinces and can infect various mammals, including blue foxes, pigs, horses, cows, and sheep. The detection of antibodies against GETV in febrile patients and its ability to infect human and primate cells highlight its potential public health risk. In pigs, GETV infections can cause severe symptoms such as arthritis, fever, anorexia, diarrhea, tremors, hind limb paralysis, ataxia, and high mortality in piglets, as well as reproductive disorders in pregnant sows. Continuous outbreaks since 2015 have led to significant economic losses in the pig industry due to the lack of effective vaccines or treatments. Enhancing early detection and surveillance of GETV is crucial for the diagnosis, prevention, and control of the disease.

Several detection tools, including RT-PCR, RT-qPCR, LAMP, RAA, and ELISA, have been developed for GETV. However, these methods are often limited by expensive instruments and complex processes, making them unsuitable for rapid, portable clinical detection. The P*f*Ago system offers a novel approach for nucleic acid molecular diagnostics, providing very fast, high specificity and accuracy by preventing false positive results through step-by-step cleavage specificity. The LAMP-P*f*Ago assay developed in this study has a minimum detection limit of 10^2^ copies/µL and no cross-reactivity with other swine-associated pathogens. The results can be observed directly with the naked eye, greatly simplifying the detection procedures. This method provides a rapid, accurate, efficient, portable, and low-cost diagnostic tool for GETV, which could be a valuable and powerful tool for the field detection and control of the virus.

## Figures and Tables

**Table 1:** Sequence of primers, gDNAs, and probes used in this study.

Name	Sequence (5′-3′)
GETV-FIP	GGATGGTGACGCCTGTTGGAACAGCATTTTCGCATCTGGCTA
GETV-BIP	CACCTACCACTGCATCTGCCCAGCTTTCGAGCGTAATTCGC
GETV-F3	ACAGCAGGTCACACCGAA
GETV-B3	CAGTCCCCGATGCTTTCG
GETV-LB	GAAAAGTGCGGAAGACCCAGA
gDNA1	TGGTCAGACATCAACC
gDNA2	TCCTTGCGGGTGCACT
gDNA3	GGGTAGTGCACCCGCA
probe	6-FAM-TCAACCTCCTTGCGGGT-BHQ1
GETV-NSP1-F	ATGGCGGACGTGTGACATCA
GETV-NSP1-R	CACAGCGTATACGTCCTGGT
qPCR-F	AGCATTTTCGCATCTGGCTAC
qPCR-R	TCTGGGTCTTCCGCACTTTT

**Table 2:** Comparison of clinical sample detection results between RT-LAMP-P*f*Ago and RT-qPCR methods.

Results	RT-qPCR (Positive/Total)	RT-LAMP-P*f*Ago (Positive/Total)
Number of positive samples	70/86	70/86
Positive rate	81.4%	81.4%

## Data Availability

Data is contained within the article.

## References

[1] Li Y.Y., Liu H., Fu S.H., Li X.L., Guo X.F., Li M.H., Feng Y., Chen W.X., Wang L.H., Lei W.W. (2017). From discovery to spread: The evolution and phylogeny of Getah virus. Infect. Genet. Evol..

[2] Chen R., Mukhopadhyay S., Merits A., Bolling B., Nasar F., Coffey L.L., Powers A., Weaver S.C. (2018). Ictv Report Consortium. ICTV Virus Taxonomy Profile: Togaviridae. J. Gen. Virol..

[3] Wang N., Zhai X., Li X., Wang Y., He W.T., Jiang Z., Veit M., Su S. (2022). Attenuation of Getah Virus by a Single Amino Acid Substitution at Residue 253 of the E2 Protein that Might Be Part of a New Heparan Sulfate Binding Site on Alphaviruses. J. Virol..

[4] Li B., Wang H., Liang G. (2022). Getah Virus (Alphavirus): An Emerging, Spreading Zoonotic Virus. Pathogens.

[5] Kanamitsu M., Taniguchi K., Urasawa S., Ogata T., Wada Y., Wada Y., Saroso J.S. (1979). Geographic distribution of arbovirus antibodies in indigenous human populations in the Indo-Australian archipelago. Am. J. Trop. Med. Hyg..

[6] Sanderson C.J. (1969). A serologic survey of Queensland cattle for evidence of arbovirus infections. Am. J. Trop. Med. Hyg..

[7] Shortridge K.F., Mason D.K., Watkins K.L., Aaskov J.G. (1994). Serological evidence for the transmission of Getah virus in Hong Kong. Vet. Rec..

[8] Sugiyama I., Shimizu E., Nogami S., Suzuki K., Miura Y., Sentsui H. (2009). Serological survey of arthropod-borne viruses among wild boars in Japan. J. Vet. Med. Sci..

[9] Li Y., Fu S., Guo X., Li X., Li M., Wang L., Gao X., Lei W., Cao L., Lu Z. (2019). Serological Survey of Getah Virus in Domestic Animals in Yunnan Province, China. Vector Borne Zoonotic Dis..

[10] Zhao J., Dellicour S., Yan Z., Veit M., Gill M.S., He W.T., Zhai X., Ji X., Suchard M.A., Lemey P. (2023). Early Genomic Surveillance and Phylogeographic Analysis of Getah Virus, a Reemerging Arbovirus, in Livestock in China. J. Virol..

[11] Shi N., Zhu X., Qiu X., Cao X., Jiang Z., Lu H., Jin N. (2022). Origin, genetic diversity, adaptive evolution and transmission dynamics of Getah virus. Transbound. Emerg. Dis..

[12] Kamada M., Wada R., Kumanomido T., Imagawa H., Sugiura T., Fukunaga Y. (1991). Effect of viral inoculum size on appearance of clinical signs in equine Getah virus infection. J. Vet. Med. Sci..

[13] Yang T., Li R., Hu Y., Yang L., Zhao D., Du L., Li J., Ge M., Yu X. (2018). An outbreak of Getah virus infection among pigs in China, 2017. Transbound. Emerg. Dis..

[14] Shibata I., Hatano Y., Nishimura M., Suzuki G., Inaba Y. (1991). Isolation of Getah virus from dead fetuses extracted from a naturally infected sow in Japan. Vet. Microbiol..

[15] Shi N., Liu H., Li L.X., Hu B., Zhang L., Zhao C.F., Deng X.Y., Li X.T., Xue X.H., Bai X. (2018). Development of a TaqMan probe-based quantitative reverse transcription PCR assay for detection of Getah virus RNA. Arch. Virol..

[16] Nie M., Deng H., Zhou Y., Sun X., Huang Y., Zhu L., Xu Z. (2021). Development of a reverse transcription recombinase-aided amplification assay for detection of Getah virus. Sci. Rep..

[17] Liu H., Li L.X., Bu Y.P., Liu Y., Sun X.T., Shi N., Deng X.Y., Lu R.G., Hu B., Jin N.Y. (2019). Rapid Visual Detection of Getah Virus Using a Loop-Mediated Isothermal Amplification Method. Vector Borne Zoonotic Dis..

[18] Sun Q., Xie Y., Guan Z., Zhang Y., Li Y., Yang Y., Zhang J., Li Z., Qiu Y., Li B. (2022). Seroprevalence of Getah virus in Pigs in Eastern China Determined with a Recombinant E2 Protein-Based Indirect ELISA. Viruses.

[19] Liu H., Zhang X., Li L.X., Shi N., Sun X.T., Liu Q., Jin N.Y., Si X.K. (2019). First isolation and characterization of Getah virus from cattle in northeastern China. BMC Vet. Res..

[20] Qiu X., Cao X., Shi N., Zhang H., Zhu X., Gao Y., Mai Z., Jin N., Lu H. (2022). Development and application of an indirect ELISA for detecting equine IgG antibodies against Getah virus with recombinant E2 domain protein. Front. Microbiol..

[21] Liu H., Hu J., Li L.X., Lu Z.S., Sun X.T., Lu H.J., Jin N.Y., Zhang L., Zhang L.N. (2023). Seroepidemiological investigation of Getah virus in the China-Myanmar border area from 2022–2023. Front. Microbiol..

[22] Cao X., Qiu X., Shi N., Ha Z., Zhang H., Xie Y., Wang P., Zhu X., Zhao W., Zhao G. (2022). Establishment of a reverse transcription real-time quantitative PCR method for Getah virus detection and its application for epidemiological investigation in Shandong, China. Front. Microbiol..

[23] Wang Z., Chen H., Hu A., Cui X., Shi C., Lu Z., Meng F., Lv F., Zhao H., Bie X. (2024). Establishment of LAMP-CRISPR/Cas12a for rapid detection of Escherichia coli O157:H7 and one-pot detection. Food Microbiol..

[24] Kropocheva E.V., Lisitskaya L.A., Agapov A.A., Musabirov A.A., Kulbachinskiy A.V., Esyunina D.M. (2022). Prokaryotic Argonaute Proteins as a Tool for Biotechnology. Mol. Biol..

[25] Swarts D.C., Hegge J.W., Hinojo I., Shiimori M., Ellis M.A., Dumrongkulraksa J., Terns R.M., Terns M.P., van der Oost J. (2015). Argonaute of the archaeon *Pyrococcus furiosus* is a DNA-guided nuclease that targets cognate DNA. Nucleic Acids Res..

[26] Kim S.Y., Jung Y., Lim D. (2020). Argonaute system of Kordia jejudonensis is a heterodimeric nucleic acid-guided nuclease. Biochem. Biophys. Res. Commun..

[27] He R., Wang L., Wang F., Li W., Liu Y., Li A., Wang Y., Mao W., Zhai C., Ma L. (2019). Pyrococcus furiosus Argonaute-mediated nucleic acid detection. Chem. Commun..

[28] Ye X., Zhou H., Guo X., Liu D., Li Z., Sun J., Huang J., Liu T., Zhao P., Xu H. (2022). Argonaute-integrated isothermal amplification for rapid, portable, multiplex detection of SARS-CoV-2 and influenza viruses. Biosens. Bioelectron..

[29] Zhao Y., Zhou C., Guo B., Yang X., Wang H. (2024). Pyrococcus furiosus Argonaute-mediated porcine epidemic diarrhea virus detection. Appl. Microbiol. Biotechnol..

[30] Zhao Y., Zhang T., Zhou C., Guo B., Wang H. (2024). Pyrococcus furiosus Argonaute Based Detection Assays for Porcine Deltacoronavirus. ACS Synth. Biol..

[31] Han R., Wang F., Chen W., Ma L. (2024). A Fast and Sensitive One-Tube SARS-CoV-2 Detection Platform Based on RTX-PCR and Pyrococcus furiosus Argonaute. Biosensors.

[32] Liu Y., Xia W., Zhao W., Hao P., Wang Z., Yu X., Shentu X., Sun K. (2023). RT-RPA-PfAgo System: A Rapid, Sensitive, and Specific Multiplex Detection Method for Rice-Infecting Viruses. Biosensors.

[33] Zhou X., Zhang T., Song D., Huang T., Peng Q., Chen Y., Li A., Zhang F., Wu Q., Ye Y. (2017). Comparison and evaluation of conventional RT-PCR, SYBR green I and TaqMan real-time RT-PCR assays for the detection of porcine epidemic diarrhea virus. Mol. Cell Probes..

[34] Rattanatumhi K., Prasertsincharoen N., Naimon N., Kuwata R., Shimoda H., Ishijima K., Yonemitsu K., Minami S., Supriyono, Tran N.T.B. (2022). A serological survey and characterization of Getah virus in domestic pigs in Thailand, 2017–2018. Transbound. Emerg. Dis..

[35] Lu G., Ou J., Ji J., Ren Z., Hu X., Wang C., Li S. (2019). Emergence of Getah Virus Infection in Horse with Fever in China, 2018. Front. Microbiol..

[36] Guth S., Hanley K.A., Althouse B.M., Boots M. (2020). Ecological processes underlying the emergence of novel enzootic cycles: Arboviruses in the neotropics as a case study. PLoS Negl. Trop. Dis..

[37] Liu Y., Chen L., Zhang Z., Zhang R., Xu J., Yang P., Sun Y., Chen Y., Xie C., Lin M. (2024). Development and application of a novel recombinase polymerase amplification-Pyrococcus furiosus argonaute system for rapid detection of goose parvovirus. Poult. Sci..

[38] Yu Z., Shi D., Dong Y., Shao Y., Chen Z., Cheng F., Zhang Y., Wang Z., Tu J., Song X. (2024). Pyrococcus furiosus argonaute combined with loop-mediated isothermal amplification for rapid, ultrasensitive, and visual detection of fowl adenovirus serotype 4. Poult. Sci..

[39] Xun G., Liu Q., Chong Y., Guo X., Li Z., Li Y., Fei H., Li K., Feng Y. (2021). Argonaute with stepwise endonuclease activity promotes specific and multiplex nucleic acid detection. Bioresour. Bioprocess..

[40] Zhao C., Yang L., Zhang X., Tang Y., Wang Y., Shao X., Gao S., Liu X., Wang P. (2022). Rapid and Sensitive Genotyping of SARS-CoV-2 Key Mutation L452R with an RPA-PfAgo Method. Anal. Chem..

[41] Zhao Y., Zhang Y., Wu W., Kang T., Sun J., Jiang H. (2024). Rapid and sensitive detection of *Mycoplasma synoviae* using RPA combined with *Pyrococcus furiosus* Argonaute. Poult. Sci..

